# Using Crowdsourcing to Evaluate Published Scientific Literature: Methods and Example

**DOI:** 10.1371/journal.pone.0100647

**Published:** 2014-07-02

**Authors:** Andrew W. Brown, David B. Allison

**Affiliations:** Office of Energetics, Nutrition Obesity Research Center, School of Public Health, University of Alabama at Birmingham, Birmingham, Alabama, United States of America; Université de Montréal, Canada

## Abstract

Systematically evaluating scientific literature is a time consuming endeavor that requires hours of coding and rating. Here, we describe a method to distribute these tasks across a large group through online crowdsourcing. Using Amazon's Mechanical Turk, crowdsourced workers (microworkers) completed four groups of tasks to evaluate the question, “Do nutrition-obesity studies with conclusions concordant with popular opinion receive more attention in the scientific community than do those that are discordant?” 1) Microworkers who passed a qualification test (19% passed) evaluated abstracts to determine if they were about human studies investigating nutrition and obesity. Agreement between the first two raters' conclusions was moderate (κ = 0.586), with consensus being reached in 96% of abstracts. 2) Microworkers iteratively synthesized free-text answers describing the studied foods into one coherent term. Approximately 84% of foods were agreed upon, with only 4 and 8% of ratings failing manual review in different steps. 3) Microworkers were asked to rate the perceived obesogenicity of the synthesized food terms. Over 99% of responses were complete and usable, and opinions of the microworkers qualitatively matched the authors' expert expectations (e.g., sugar-sweetened beverages were thought to cause obesity and fruits and vegetables were thought to prevent obesity). 4) Microworkers extracted citation counts for each paper through Google Scholar. Microworkers reached consensus or unanimous agreement for all successful searches. To answer the example question, data were aggregated and analyzed, and showed no significant association between popular opinion and attention the paper received as measured by Scimago Journal Rank and citation counts. Direct microworker costs totaled $221.75, (estimated cost at minimum wage: $312.61). We discuss important points to consider to ensure good quality control and appropriate pay for microworkers. With good reliability and low cost, crowdsourcing has potential to evaluate published literature in a cost-effective, quick, and reliable manner using existing, easily accessible resources.

## Introduction

The evaluation of published research literature requires human intelligence to complete (so called Human Intelligence Tasks, or HITs) and is therefore difficult or impossible to automate by computer. Yet, many of the individual tasks that make up a typical evaluation (e.g., extraction of key information, coding of qualitative data) may be amenable to crowdsourcing, the practice of dividing a large task across an often disjointed group of individuals. Crowdsourcing has been used in other contexts to evaluate such complex topics as codifying sleep patterns [1], classifying biomedical topics [2], and creating speech and language data [3]. Herein, our primary purpose is to examine the feasibility of using crowdsourcing to evaluate scientific literature. To do so, we use the following question as an example: “Do nutrition-obesity studies with conclusions concordant with popular opinion receive more attention in the scientific community than do those that are discordant?” If conclusions that are concordant with popular opinion are afforded more attention by the scientific community (i.e., higher citation counts or published in a higher-ranked journal), it may be evidence of distortion of the scientific record. This question requires four kinds of HITs: 1) categorization of study abstracts; 2) iterative refinement of free-text responses; 3) eliciting subjective opinions; and 4) a simple data extraction of citation counts. We will first describe the crowdsourcing platform, followed by discussing each of these HITs, and then use the data to examine the example question. We conclude the narrative with additional discussion and remarks about crowdsourcing, followed by a more in depth description of the methodology.

## Method Narrative and Evaluation

**Figure 1:**
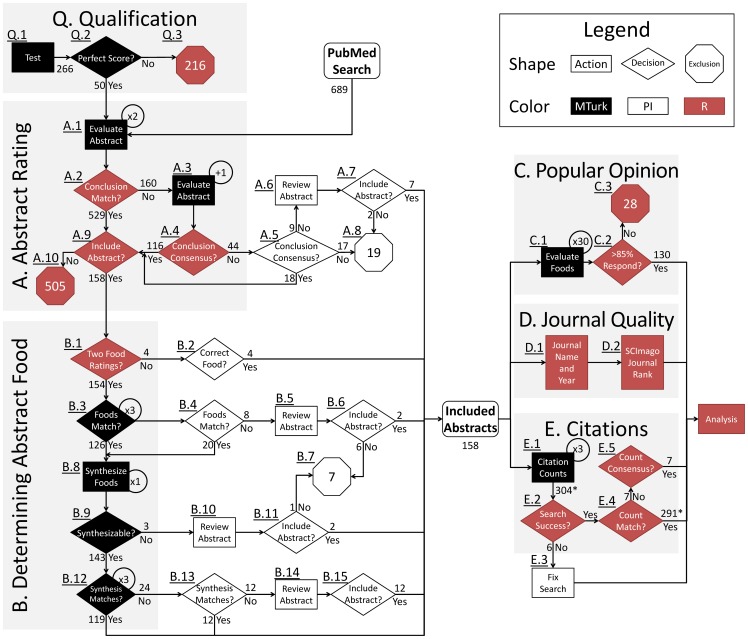
Flow chart of crowdsourcing procedures. Steps are separated into sections based on separate tasks. The more granular of a process that can be made, the more amenable the process is to crowdsourcing. MTurk: process completed on MTurk. PI: process completed by AWB. R: process completed with custom R scripts. Gray boxes include tasks automated through R or MTurk, while steps outside of the gray boxes were manually completed. Circled numbers represent the number of times a task was completed. In box E, 304 preliminarily included abstracts (including the 158 ultimately included) had citation counts extracted while the final abstract ratings were concurrently being completed from box A.

We chose to use Amazon's Mechanical Turk (MTurk; www.mturk.com) crowdsourcing platform. MTurk is a flexible, online, task-based, microwork marketplace in which crowdsourcing workers (microworkers) can choose to accept tasks posted by requesters (in this case, AWB and DBA) for a fee defined by the requester. The requester can set qualification criteria (e.g., self-reported age and location; MTurk aggregated work history; custom-defined qualifications), and choose whether to accept or reject work after it has been submitted before paying. Freely available MTurk interfaces allow the creation of common digital question formats (e.g., list box, multiple choice, free-text) with more formats available through custom coding. Considerations for each step in our workflow (Fig. 1) include: 1) how involved our non-crowdsourced research team (AWB and DBA) would be in evaluating questions; 2) how qualified each microworker needed to be; 3) how to phrase questions and tasks such that HITs could be completed by the targeted microworkers; and 4) how many times each question needed to be evaluated to expect reliable responses.

**Figure 2:**
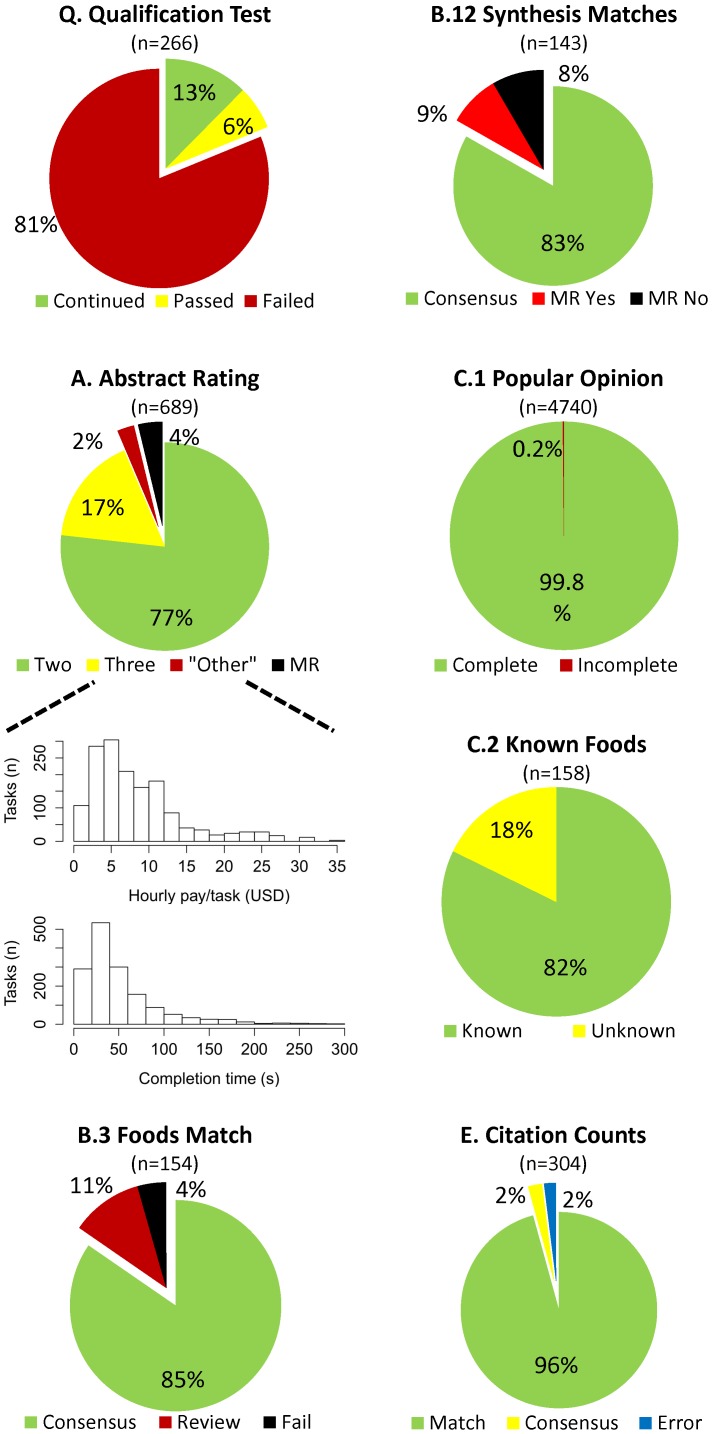
Performance metrics of microworkers. In each chart, green  =  desirable, yellow  =  acceptable, red  =  undesirable, blue  =  issue external to crowdsourcing, and black  =  failure. The letters preceding each chart title corresponds to the steps in Fig. 1, and the number in parentheses represents the number of units compared. **A**) Two: consensus after two ratings; Three: consensus after three ratings; “Other”: consensus after reviewing free-text answers; MR: manually reviewed by AWB. The accompanying histograms show the distributions of hourly pay calculated by completion time, and completion time in seconds for each task. **B.3**) Review: microworkers did not agree the foods matched, AWB reviewed the foods extracted from abstracts, and they matched; Fail: the foods did not match after review from AWB. **B.12**) MR Yes: AWB determined the synthesized term matched the original two foods; MR No: AWB determined the synthesized term did not match the original two foods. **C.1**) Popular opinion questions were either completely answered or not. **C.2**) Known: less than 15% did not know the food; Unknown: greater than 15% did not know the food. **E**) Match: 3 ratings matched; Consensus: 2 of 3 matched; Error: the link provided to the microworkers was faulty.

The first set of HITs (Fig. 1A) was designed to examine the feasibility of employing microworkers to categorize study abstracts. Microworkers were asked to rate 689 abstracts about nutrition and obesity to determine: 1) if the study was about humans, thereby excluding animal studies, *in vitro* studies, commentaries, and reviews (a multiple choice question); 2) whether the study investigated the relation of foods or beverages (hereafter referred to as ‘food’ for simplicity) with obesity (a multiple choice question); 3) which food was described in the paper (a free-text question); and 4) the conclusions about the relation between the food and obesity (a multiple choice question). This also limited studies to those comparing one food along a range of exposures or to a control. Microworkers were only allowed to complete these HITs if they satisfied two types of qualification. First, built-in MTurk Qualifications selected only microworkers that were United States residents and at least 18 years old. Second, a custom MTurk Qualification required microworkers to correctly categorize 3 example abstracts (Fig. 1Q and Fig. 2Q).

Agreement was reached in 529 of the 689 abstracts after two microworker assessments (77%, in Fig. 2A). Interrater agreement was moderate from these first two ratings [4] (κ = 0.528 when all free-text conclusions were categorized as ‘other;’ κ = 0.586 when excluding free-text answers). An additional 17% reached consensus after a third rater, and 2% more reached consensus when free-text answers (“Other”) were reviewed by AWB. Only 4% of abstracts had to be rated by AWB because of lack of consensus, and only 7 of the 158 abstracts coded to be included (Step A.9) were subsequently rejected in other steps (4% of the 158). The median abstract evaluation time was 38 seconds, which resulted in an estimated median wage of $6.63/hr USD as calculated from task completion time (Fig. 2A histograms).

The next set of three HITs investigated iterative refinement of free-text answers by synthesizing the free-text food-topic answers into one coherent term (Steps B.1-B.15). Three microworkers confirmed that the two food-topics reported for each abstract (e.g., “Pistachios” and “pistachio nuts”) referred to the same food (Fig. 2B.3). For topics that at least two of three microworkers said matched, a separate HIT was posted to synthesize the two foods into one coherent phrase (e.g., “pistachios”). New phrases were compared to the original phrases by two additional microworkers to confirm that they were a reasonable synthesis of the original phrases (Fig. 2B.12). If the phrases failed to match or be synthesized at any point during this quality control, AWB reviewed them (Steps B.2, B.4, and B.13), and if need-be he directly reviewed the abstract (Steps B.5, B.10, and B.14). Each assignment was awarded $0.01 USD.

**Figure 3:**
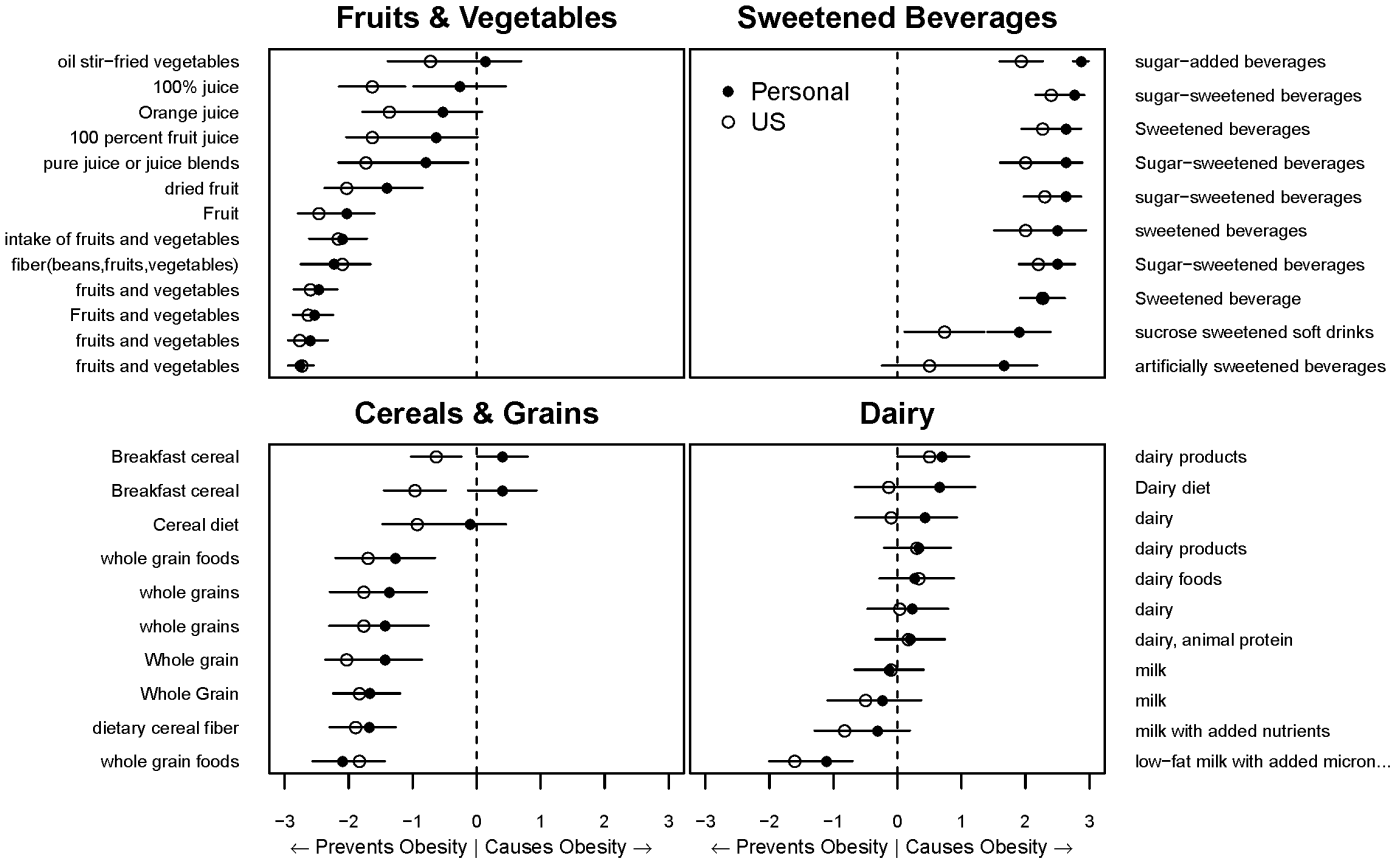
Example of groups of foods and average obesogenicity ratings. Each food listed on the y-axes is shown as synthesized by microworkers in Fig. 1 Step B.9. Foods are ordered within each panel top to bottom with the personal opinion from most to least obesogenic. The vertical dotted line represents the transition from popular opinion indicating the food prevents obesity (left) to causes obesity (right). Each point represents the mean ±95% CI. Note that these results are only meant to reflect an estimate of popular opinion, and do not necessarily reflect the actual obesogenicity of any given food.

In addition to categorization and iterative synthesis, subjective opinions can also be elicited from microworkers. Microworkers were asked to rate the perceived obesogenicity of the foods synthesized above (Step C.1) and to predict the overall US perception of the food using a 7 point, horizontal, multiple choice scale from “prevents obesity” to “causes obesity” (e.g., x-axis in Fig. 3), with an option to indicate they did not know a food. Microworkers were allowed to respond to all 158 foods, but only once for each food. Thirty responses were collected for each food at $0.01 USD each. Only 8 obesogenicity responses were unusable out of 4740 (<1%; Fig. 2C.1). These survey results are only meant to reflect an estimate of popular opinion, and do not necessarily reflect the actual obesogenicity of any given food.

Qualitative and quantitative review of the opinion answers supports internal and external consistency of microworker opinions. Most foods were known by the microworkers (Fig. 2C.2). Foods categorized as “unknown” (>15% of respondents did not know the food; Fig. S1 and Table S1) included conjugated linoleic acid (n = 5), references to glycemic index (n = 3), and specific foods such as mangosteen juice, Chungkookjang, and “Street food in Palermo, Italy;” none of these foods would be expected to be widely known in the general US population. Also, “known” and “unknown” foods comprised mutually exclusive lists. In addition, microworkers' predictions of US opinions track well with the aggregated individual opinions (Fig. 3). Finally, popular opinions seem to match our expert expectations: fruits and vegetables were rated as preventing obesity, with less certainty about fried vegetables or fruit juice, and sweetened beverages were rated as causing obesity, with artificial sweeteners considered to be less obesogenic.

A simple data extraction task was also presented to microworkers. Google Scholar search links were presented to microworkers and microworkers reported the number of times the paper was cited. Each of three ratings was awarded $0.01 USD. Only 7 papers did not have identical reported citations counts among the 304 abstracts searched (2%; Fig. 2E). All 7 of these papers had two of three responses matching, which matched a manual review by AWB.

To answer the question initially posed, the data extracted from these steps were synthesized with a measurement of journal quality (ScImago Journal Rank, SJR). As expected, the number of citations a paper received increased with journal quality (15.13±2.66 citations per unit increase of SJR, p = 6.4196e-8) and time since publication (10.26±1.32 citations per year since publication, p = 1.0187e-12). No models that tested whether opinion-conclusion concordance was associated with scientific attention were statistically significant. Specifically: log citation counts were not predicted by a continuous measurement of concordance when controlling for publication date and SJR (1.09 per unit concordance increase; 95% CI: 0.96,1.24; p = 0.2021), nor was log SJR predicted by continuous concordance (0.96 per unit concordance increase; 95% CI: 0.86,1.08; p = 0.5218); and log citation counts were not different between categorically concordant versus discordant conclusions when controlling for publication date and SJR (1.02; 95% CI: 0.78,1.33; p = 0.8823), nor was log SJR different (0.87; 95% CI: 0.69,1.10; p = 0.2454).

## Discussion

The above narrative describes the potential and feasibility of using crowdsourcing to evaluate published literature. Several important considerations must be made regarding the implementation of crowdsourcing for literature analysis or any other complex task, some of which are described in more detail in the methods section below. First and foremost, using crowdsourcing does not preclude the need for the quality control checks that would be implemented in any other data extraction or survey methodology. Indeed, when highly technical information is being acquired through crowdsourcing, quality checks are essential to not only guarantee the validity of the results but also to reassure the reader that competent individuals have assembled the information. It is also important to note that microworkers self-certify their age and location, which is a common limitation of any self-reported results. Using HITs stored outside of MTurk can provide the potential for IP address confirmation, which is a more reliable confirmation of location; we did not use such methodology herein. Investigators must determine what level of confidence they need in the identities of respondents. For technical tasks, such as literature evaluation, qualification tests like the ones we employed can be used to help guarantee competency, which in this example was more important than identity. Finally, it is important for investigators to consider the desired scope of the literature evaluation. In this case-study, our inclusion/exclusion methods may have resulted in papers being excluded by chance early that should have been included, but the final corpus should not have included papers that should have been excluded. It was more important for our example that the final corpus only include studies of interest than to have an exhaustive inclusion; this is clearly not the case for someone interested in conducting a comprehensive systematic review, and thus other controls would need to be included in such cases.

There is also an upfront cost for investigators to become familiar with the infrastructure that is not calculated into the cost of microworkers reported above. For laboratories with sufficient programming knowledge (e.g., SOAP and REST requests, HTML, Java, XML, or command line), adapting the existing MTurk infrastructure should be fairly straightforward. Similarly, for simple data-extraction tasks where an objective, easy-to-understand answer to the task exists (in our example, citation counts), applying quality control to the results is also straightforward. In other circumstances, researchers must weigh the time it takes to become familiar with and implement a new method versus the time it takes to complete the tasks with existing methodology. Time and cost savings will depend on the difficulty of the task, the difficulty in validating microworker responses, the size of the task, and the prospects of continued use of the methods by the research group. We are presently working to address some of the barriers other groups may encounter when attempting to implement crowdsourcing for literature evaluation.

The amount of money paid for each task must also be carefully considered. Because resources such as MTurk are microwork marketplaces, the quality of work, the number of microworkers attracted to a task, and the timeliness of task completion may be related to how much is paid. Just as important is the fairness of pay for the microworkers. The total cost paid to microworkers in these tasks was $221.75 USD. In our abstract evaluations, the extrapolated median hourly pay for the abstract evaluation task was $6.63 USD, which was marginally lower than our target ($7.25 USD, the United States' minimum wage). The overall median hourly pay across all tasks was $5.14 USD (Fig. S2). Although each assignment a microworker accepts is, in effect, a form of microcontract work and is therefore, as we understand it, not subject to minimum wage laws, the ethics of having an individual working at a rate below established minimum pay is questionable. Inflating our $221.75 estimate to have a median hourly pay of $7.25 USD, the expected cost of tasks in this project would have been $312.61. We have identified several areas *post hoc* that we and others may want to consider to better ensure fair pay. 1) Calculate pay rates based on individuals that have completed at least several tasks. We noted that the first few tasks an individual completes are typically slower, so a group of individuals that completes few tasks will inflate the amount of time it takes to complete a task, which will unfairly raise the cost of the tasks for the requester. 2) Calculate pay rates by discarding particularly rapid completers. Although investigators want work completed rapidly and accurately, calculating pay rates based on the most rapid individuals may effectively deflate the pay for average, but otherwise good, microworkers. 3) Estimate time-to-completion *a priori* by having an investigator complete the task in the MTurk Sandbox. This will allow the investigator to include the amount of time it takes for the webpages to load and submissions to be processed, which is included in the calculated time it takes to complete a task. Webpage load time is likely the greatest influence on our average pay rate for the $0.01 tasks because webpages can take several seconds to load. Decreasing the load time for the task can increase an investigator's return on investment and microworkers' pay. 4) If the final rate target was missed, give bonuses to individuals completing some threshold of tasks to at least average minimum wage.

## Conclusions

The value of science is dependent on complete and transparent reporting of scientific investigations, but recent examples highlight the existence of incomplete or distorted research reporting [5], [6]. Here, we have demonstrated that the careful use of crowdsourcing can be an economical and timesaving means to evaluate large bodies of published literature. Manually and continuously evaluating literature may be manageable for specific subtopics, but may be unwieldy even for a topic as specific as obesity (>18,000 papers in PubMed indexed in 2012 alone). Crowdsourcing can save calendar time by increasing the number of individuals working on a given task at one time. Because many of the steps in study preparation (e.g., coding sheets; literature searches) and calculations (e.g., interrater reliability; hypothesis testing) are analogous to those of traditional in-house evaluation methods, the time investment from appropriately trained research groups should not be substantially greater. With good reliability and an estimated total microworker cost of $312.61 for the tasks evaluated herein, appropriately constructed and controlled crowdsourcing has potential to help improve the timeliness of research evaluation and synthesis.

## Methods

### Human subjects approval

This work was approved under Exemption Status by the University of Alabmama at Birmingham's Institutional Review Board (E130319007). Each HIT included a highlighted statement that informed the microworkers that the task was being used as part of a research project, and by accepting the HIT they certified that the microworker was at least 19 years old. Contact information for AWB and the University of Alabama at Birmingham IRB were provided with this statement.

### Interfacing with MTurk

HITs were constructed using HTML and Java, using the Mechanical Turk Command Line Tools (version 1.3.1) and the Java API (version 1.6.2).

### Search

PubMed was searched for abstracts of human studies about foods and beverages (hereafter referred to simply as food) and obesity that were not reviews: obesity [majr] AND food [majr] NOT review [ptyp] AND humans [mh] AND English [lang], where abstracts were available and date range 2007-01-01 to 2011-12-31. Because public opinion about foods may drift through time, the abstract date range was limited; in addition, ending the search at the end of 2011 allowed time for the articles to be cited. Abstracts from 689 papers were considered.

### Microworker Qualifications

We restricted microworkers to United States residents over 18 years of age using built-in MTurk qualification requirements. Because the age of majority is 19 in the state of Alabama, microworkers were informed in the HIT description and the IRB header on each HIT that they needed to be older than 19. Age and location restrictions are only confirmed by self-certification. To complete HITs for the initial categorization of abstracts (Fig. 1, Step A.1), microworkers had to complete a qualification test including three example abstracts (Step Q.1). The abstracts used for the qualification test were related to, but not included in, the 689 abstracts to be categorized. Microworkers had to complete the qualification with 100% accuracy to proceed to categorize abstracts, and were not allowed to retake the qualification test (Step Q.2). The other tasks (B, C, and E) were simple and straight forward, so only the residency and age restriction qualifications were enforced.

### Abstract Rating

A web form with the PubMed abstract page embedded in an HTML iFrame was presented to microworkers who elected to complete the sorting and successfully completed the qualification test. The response tools available in the Command Line Tools and Java API reflect standard web-based response tools, including multiple choice, checkbox, list selection, and free-text, among others. This HIT used a mixture of these by asking microworkers to determine: 1) if the study was about humans, which was meant to exclude animal studies, *in vitro* studies, commentaries, and reviews (multiple choice); 2) if the study investigated the relation of foods with obesity (multiple choice); 3) what food was described in the paper (free-text answer); and 4) the conclusions about the food and obesity (multiple choice). Responses had to be internally consistent. For example, an abstract could not be rated as being a non-human study and then have conclusions about the food and obesity given. Only 10 of 1538 responses (<1%) had internal consistency issues.

If two microworkers concurred about the conclusions (Step A.2), then the abstract was passed to the next phase (Step A.9). Some rating disagreements were the result of microworkers typing answers into the provided free-text ‘other’ box rather than selecting an option. When disagreements existed, the abstract was evaluated a third time (Step A.3). The abstract was passed to the next phase (Step A.4-A.9) if at least 2 of 3 ratings agreed. Otherwise, the abstract was manually rated by AWB (Steps A.5-A.8). Each abstract rating received $0.07.

Kappa statistics are typically calculated with raters rating each item in a set. Because microworkers did not rate each item within a task, a kappa-type statistic (κ in the text) was calculated by ordering raters based on the number of items a microworker completed within each task. Within each item, the most prolific microworker was designated rater 1 and the next most prolific microworker as rater 2. Interrater agreement was calculated by Conger's extension of Cohen's kappa [7] using the ‘exact’ option of the kappam.fleiss function of R package “irr” version 0.84.

### Determining Abstract Food Topic

The iterative synthesis was described in the narrative text.

### Popular Opinion About Foods

In Step C.1, microworkers were asked their opinions on the perceived obesogenicity of foods identified in Step B. Opinion questions of this nature are subject to the same concerns as survey or opinion questions evaluated in other settings (e.g., [8]). Microworkers were told to evaluate on a 7 point, horizontal, multiple choice scale whether a food “prevents obesity” or “causes obesity”. In an effort to prevent microworkers from overstating their personal beliefs to influence a perceived counter belief of others, they were also asked to predict whether most Americans thought the food “prevents obesity” or “causes obesity”. Microworkers were encouraged to click an option indicating they did not know what a food was rather than looking up information or guessing. HITs were posted on 3 different days and times to include a variety of microworkers and microworkers were allowed to respond to all 158 foods, but only once for each food. Thirty responses were collected for each food at $0.01 each.

Foods were categorized as “known” if >85% of respondents ranked the food, and “unknown” if >15% of respondents marked that they did not know the food (Fig. S1 for cutoffs; Table S1 for the list of “known” and “unknown” foods). Known foods were included in subsequent analyses. Figure 3 shows the ratings of related known foods.

### Journal Quality

Publication dates were extracted using custom R scripts to first look for an Epub year and month in PubMed (Step D.1); if not, year and month of the journal issue was extracted; if only a year was available, the PD was set as January 1 of that year. SCImago Journal Rank (SJR) was used as a measurement of journal quality (http://www.scimagojr.com/journalrank.php), accessed: 13 Aug 2013]. SJR's were extracted for the year prior to the PD to estimate the quality of the journal around the time the authors would have submitted an article (Step D.2).

### Citations

Google Scholar search links were presented to microworkers for each title instead of embedding in iFrames because Google does not allow their material to be placed in iFrames. Links were generated from simple concatenation of “http://scholar.google.com/scholar?q=” and URL-encoded article titles. Microworkers followed the link and reported the number of times the paper was cited on 3 June 2013 (Step E.1). Although Google Scholar includes some citation sources that are not classically considered relevant to academic circles and may miss others, it is a free, stable alternative to commercial sources [9]. 304 papers (including all papers in the final evaluation) were reviewed for citation counts. If the search failed (e.g., because of special characters in the title), citation counts were obtained by AWB (Step E.3). Reported citation counts were tested for verbatim matching (Step E.4) or consensus (Step E.5). Each of three ratings was awarded $0.01 USD.

### Model Analysis

**Figure 4:**
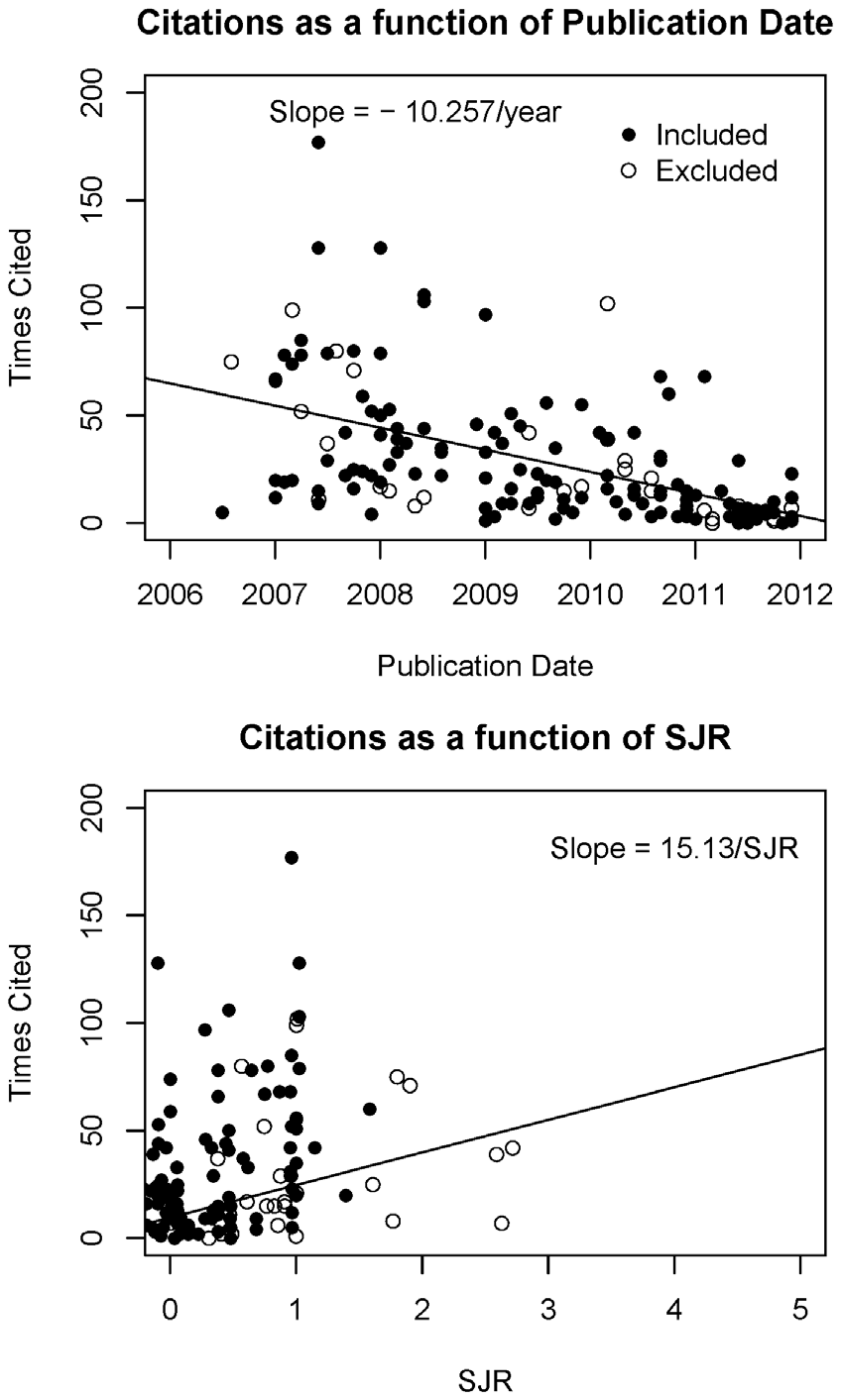
Citations increase with journal quality and time since publication. To confirm that extracted citation counts, journal rank, and publication date conformed to expected patterns, citation counts were fit as a function of publication date controlling for journal quality (SJR; upper panel) and citation counts were fit as a function of journal quality (lower panel). No differences were seen between papers that were “known” (included) and “unknown” (excluded).

**Figure 5:**
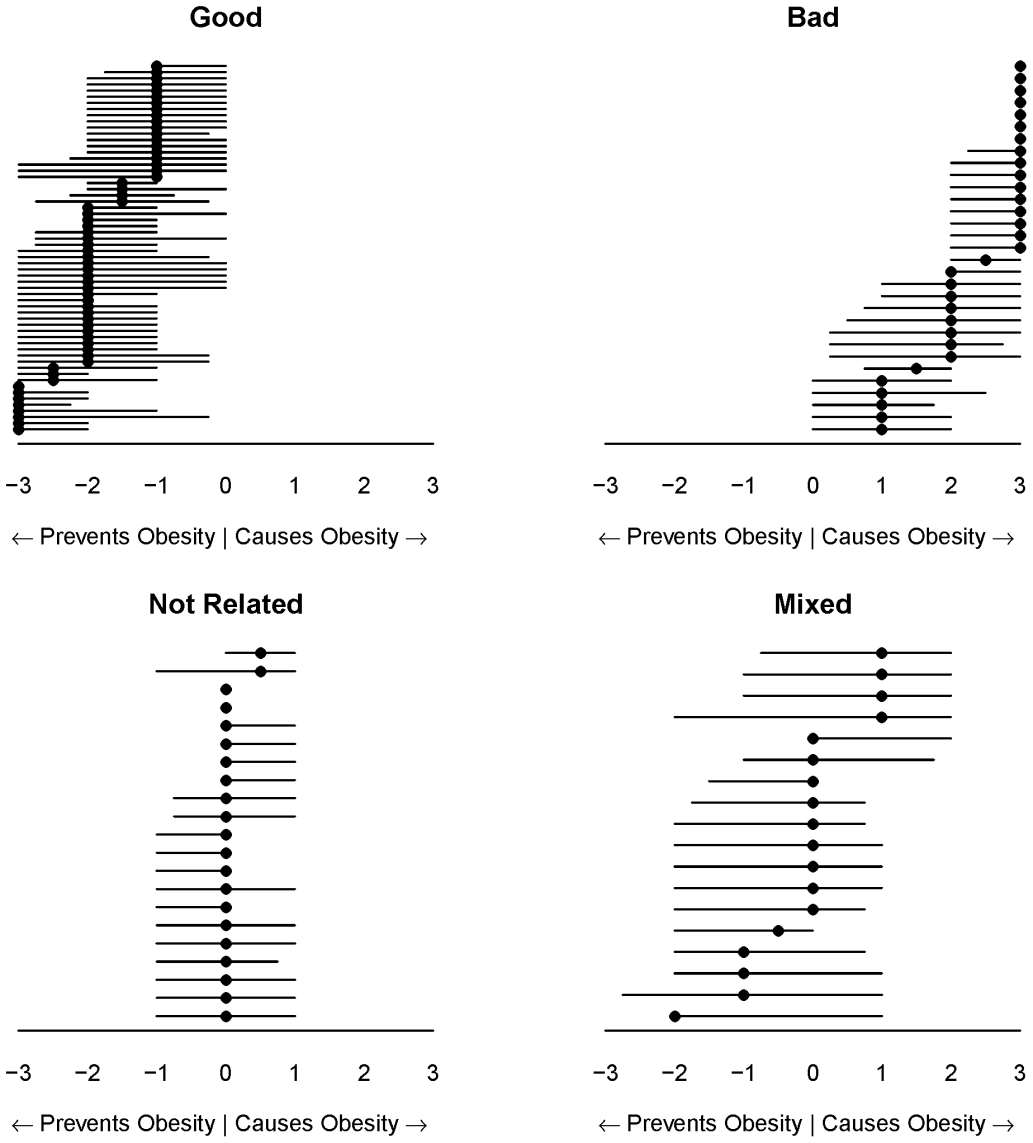
Quartile-based categorization of perceived obesogenicity foods. Foods were categorized dependent on the median and quartiles as follows: Good (“Prevents Obesity”), median ≤−1 and Q3≤0; Bad (“Causes Obesity”), median ≥1 and Q1≥0; Not Related, median and IQR between −1 and 1; Mixed (depends on who eats the food), median and IQR spanning other categories. Each dot represents the median spanned by Q1 to Q3 for each included food.

Linear models were fitted to confirm the expectation that 1) citation counts decrease with more recent publication date; and 2) citation counts increase with increasing SJR (Fig. 4). Models were also fitted to test whether natural-log-transformed (log) citation counts were associated with abstract conclusions, controlling for publication date and log SJR, and whether log SJR was associated with abstract conclusions. Two separate models were fit to test whether log citation counts or log SJR were associated with conclusions agreeing with popular opinion. In Model 1, the concordance of abstract conclusions with microworker food obesogenicity opinions were rated continuously on a scale of −3 to 3, limited only to papers that concluded the food was beneficial or detrimental for obesity. In Model 2, abstract conclusions were categorized as agreeing or disagreeing with microworker food obesogenicity opinions using quartiles as follows (Fig. 5):

- Beneficial: at least 75% of responses were less than or equal to 0, and at least 50% were less than or equal to −1.
- Detrimental: at least 75% of responses were greater than or equal to 0, and at least 50% were greater than or equal to 0.
- Not Related: the median had to be less than or equal to 1 and greater than or equal to −1, with the interquartile range also within that range.
- Unclearly Related: broad interquartile ranges not included in the definitions above.

These categorizations were then dichotomized as either matching or not matching the conclusions of the abstract. This dichotomization was used to predict log citation counts, correcting for publication date and log SJR; or to predict log SJR directly.

Variables were extracted and compared from the MTurk output using custom scripts in R. Statistical models were calculated using the glm function with default options in R version 3.0.1 using RStudio on a 64-bit Windows 7 machine.

## Supporting Information

Figure S1 **The proportion of microworkers knowing each food.** Complete answers are those where a response was given for both the US and personal opinions, or the respondent indicated they did not know what the food was. Most foods were known and rated by all microworkers as demarked by the large bar at 1.0 on the x-axis. The proportion of people knowing a food decreased until a natural break at 0.85 (vertical dashed line), which was chosen as the cutoff between classifying a food as “known” or “unknown”.
(TIFF)

Figure S2 **Median extrapolated hourly pay for microworkers.** Median hourly pay for each microworker on a given task was calculated by dividing 3600 seconds by their median completion time in seconds and multiplying by the reward amount. For task A, this amount was $0.07 USD; for tasks B.3, B.8, B.12, C, and E, the amount was $0.01. The solid horizontal line represents minimum wage ($7.25 USD); the dashed horizontal line represents median extrapolated pay rate across all tasks ($5.14 USD). Each circle represents one microworkers' median extrapolated pay rate; the area of the circle is proportional to the number of assignments a microworker completed within a HIT. The same microworkers did not necessarily work on all HITs.
(TIFF)

Table S1 **Foods from abstracts as isolated by microworkers.**
(DOCX)
