# Supplementary material for: Using Crowdsourcing to Evaluate Published Scientific Literature: Methods and Example
Source: PLoS One. 2014 Jul 2;9(7):e100647. doi: 10.1371/journal.pone.0100647 (PMC4079692; doi:10.1371/journal.pone.0100647)
Supplement: Table S1 — Foods from abstracts as isolated by microworkers. (DOCX) [file pone.0100647.s003.docx]

## **Table S1.** Foods from abstracts as isolated by microworkers

| **Known (<15% rated unknown)** | **Unknown (>15% rated unknown)** |
| --- | --- |
| 100 percent fruit juice | Chungkookjang |
| 100% juice | CLA |
| apples and pears | CLA |
| artificially sweetened beverages | CLA |
| Available fast food | conjugated linoleic acid |
| Baby formula | conjugated linoleic acid and safflower oil |
| bread | DASH diet |
| Breakfast cereal | Delayed solid food in infants |
| Breakfast cereal | diet with reduced glycemic index |
| breast milk | Energy-restricted high-protein diet with a low glycemic index and soluble fiber |
| breastfeeding | foods with low glycemic index |
| Calorie-restricted cod-based diet | high-fiber diet, including psyllium |
| carbohydrates | high monounsaturated fat diet |
| Cereal diet | HND Vegatable based diet |
| chewing gum | Hypocaloric high-protein diet |
| childfood breastfeeding | legumes |
| coconut oil | Legumes |
| cod | linseed-fed animal products |
| coffee and tea | low-calorie LHCP |
| complete mediterranean meal | Mangosteen Juice |
| dairy | Medifast meal-replacement supplements combined with appetite suppressant medication |
| dairy | non-milk extrinsic (NME) sugars |
| Dairy diet | Polyunsaturated fatty acids |
| dairy foods | PRO diet |
| dairy products | Probiotics |
| dairy products | pulses and whole grains |
| dairy, animal protein | Street food in Palermo, Italy. |
| Dark chocolate and non-chocolate snacks | white bean flavor |
| dietary caffeine |  |
| dietary cereal fiber |  |
| dietary fat intake |  |
| dietary fat intake |  |
| Dietary fat intake |  |
| Dietary fiber supplement |  |
| diets |  |
| diets high in certain fats |  |
| dried fruit |  |
| evening carb meal |  |
| fast food |  |
| fat-reduced, carbohydrate-modified diet |  |
| fiber enriched diet |  |
| fiber(beans,fruits,vegetables) |  |
| fish based diet |  |
| Fish oil fatty acids |  |
| Free pre-prepared healthy meals |  |
| fried |  |
| Fruit |  |
| fruits and vegetables |  |
| fruits and vegetables |  |
| fruits and vegetables |  |
| Fruits and vegetables |  |
| herring |  |
| high-protein |  |
| high-protein diet |  |
| high-protein diet |  |
| High-protein diet |  |
| High-protein diet |  |
| High-protein low carb diet |  |
| high dietary fat |  |
| high dietary salt food |  |
| high fat diet |  |
| High protein and Fiber diet |  |
| high protein diet |  |
| high protein intake |  |
| high protein/carb diet |  |
| honey |  |
| Imported Foods |  |
| intake of fruits and vegetables |  |
| Low-calorie meal replacement formula |  |
| low-calorie, liquid protein diet |  |
| Low-carbohydrate diet |  |
| low-fat milk with added micronutrients |  |
| Low calorie soy protein meal replacement |  |
| Low carbohydrate high protein diet |  |
| low sugar |  |
| meal replacement (SlimFast) shakes |  |
| Meal replacements |  |
| meat |  |
| meat |  |
| milk |  |
| milk |  |
| milk with added nutrients |  |
| moderate-protein diet |  |
| Moderate carbohydrate, low-fat diet |  |
| mostly meat meals |  |
| natural foods |  |
| nuts |  |
| nuts |  |
| oil stir-fried vegetables |  |
| Olive oil |  |
| Olive Oil |  |
| Orange juice |  |
| pistachios |  |
| processed potato products |  |
| proportion of fat in the diet |  |
| protein |  |
| Protein-enriched meal replacement |  |
| protein formula |  |
| protein intake |  |
| pure juice or juice blends |  |
| reduced carbohydrate intake |  |
| Reduced sugar calorie free sweetner |  |
| salt and fat-reduced meat products |  |
| Snack foods |  |
| snacking |  |
| solid food |  |
| soy-based beverages |  |
| soy-protein-rich foods |  |
| sucrose sweetened soft drinks |  |
| Sugar |  |
| sugar-added beverages |  |
| sugar-sweetened beverages |  |
| sugar-sweetened beverages |  |
| Sugar-sweetened beverages |  |
| Sugar-sweetened beverages |  |
| sugar and fat |  |
| Sugar in coffee or tea |  |
| sugars |  |
| Sweetened beverage |  |
| sweetened beverages |  |
| Sweetened beverages |  |
| traditional Mongolian diet, high in processed meat, potatoes, whole milk fats, and oils |  |
| types of moderate-fat diets |  |
| Whey Protein |  |
| Whole grain |  |
| Whole Grain |  |
| whole grain foods |  |
| whole grain foods |  |
| whole grains |  |
| whole grains |  |
